# Nutritional conditions regulate transcriptional activity of SF-1 by controlling sumoylation and ubiquitination

**DOI:** 10.1038/srep19143

**Published:** 2016-01-11

**Authors:** Jiwon Lee, Dong Joo Yang, Syann Lee, Gary D. Hammer, Ki Woo Kim, Joel K. Elmquist

**Affiliations:** 1 Departments of Pharmacology and Internal Medicine, Division of Hypothalamic Research, University of Texas Southwestern Medical Center, Dallas, Texas 75390, USA; 2 Departments of Pharmacology and Global Medical Science, Institute of Lifestyle Medicine and Nuclear Receptor Research Consortium, Wonju College of Medicine, Yonsei University, Wonju, 26426, South Korea; 3 Endocrine Oncology Program, Center for Organogenesis, University of Michigan Health System, Ann Arbor, Michigan 48109, USA

## Abstract

Steroidogenic factor 1 (SF-1) is a transcription factor expressed in the ventral medial nucleus of the hypothalamus that regulates energy homeostasis. However, the molecular mechanisms of SF-1 in the control of energy balance are largely unknown. Here, we show that nutritional conditions, such as the presence or absence of serum, affect SF-1 action. Serum starvation significantly decreased hypothalamic SF-1 levels by promoting ubiquitin-dependent degradation, and sumoylation was required for this process. SF-1 transcriptional activity was also differentially regulated by nutritional status. Under normal conditions, the transcriptional activity of hypothalamic SF-1 was activated by SUMO, but this was attenuated during starvation. Taken together, these results indicate that sumoylation and ubiquitination play crucial roles in the regulation of SF-1 function and that these effects are dependent on nutritional conditions, further supporting the importance of SF-1 in the control of energy homeostasis.

Steroidogenic factor 1 (SF-1), also known as NR5A1, is a nuclear receptor (NR) that plays an important role in the development and regulation of steroidogenesis in adrenal glands and gonads^1^. Global SF-1 knockout (KO) impairs the development of the ventral medial nucleus of the hypothalamus (VMH) and the maintenance of normal body weight^2^,^3^,^4^. SF-1 is uniquely expressed in the VMH in the brain where it functions as a key regulator of energy balance^1^,^3^,^5^,^6^,^7^. VMH-specific SF-1 KO mice have increased weight gain and impaired thermogenesis in response to high-fat diet^5^, implicating a role for neuronal SF-1 in energy homeostasis. Furthermore, disruptions in leptin and PI3K (p110α) signaling in SF-1 neurons affect body-weight homeostasis and diet-induced thermogenesis^8^,^9^, while insulin receptor deletions showed improved glucose metabolism and increased leptin sensitivity when fed a high-fat diet^10^. While these studies have demonstrated a role for SF-1 in the regulation of energy homeostasis, the molecular mechanisms underlying the actions of SF-1 are not well understood.

SF-1 is a transcription factor that controls the expression of numerous downstream target genes, including several genes known to be important in body weight homeostasis, such as cannabinoid receptor 1 (CB1), brain-derived neurotrophic factor (BDNF), and corticotrophin releasing hormone receptor 2 (Crhr2)^11^,^12^,^13^,^14^,^15^. Importantly, Baba *et al.* recently showed a role for SF-1 in the regulation of glycolytic genes, supporting the notion that SF-1 may play roles not only for body weight homeostasis but also for glucose homeostasis^16^.

The transcriptional activity of SF-1 itself is regulated by a number of transcription factors and co-regulators and by post-translational modifications^15^. For instance, phosphorylation of SF-1 at Ser203 by extracellular signal-regulated kinases 1/2 (ERK1/2) or cyclin-dependent kinase 7 (CDK7) activates downstream gene transcription and recruitment of co-factors^17^,^18^,^19^. SF-1 is acetylated by general control nonderepressed 5 (GCN5) and CREB binding protein (CBP)/p300^20^,^21^. Acetylation of SF-1 increases DNA binding and stimulates transcriptional activity. On the other hand, sumoylation at Lys194 inhibits the ability of SF-1 to activate target gene expression and induces localization of SF-1 to nuclear speckles^19^,^22^. Moreover, there is evidence of crosstalk between SF-1 sumoylation and phosphorylation. Mutations of the sumoylation site at Lys194 lead to an increase in CDK7 recruitment, which is required for Ser203 phosphorylation^19^,^23^.

Many NR transcription factors are transcriptionally regulated by sumoylation^24^. In most cases, sumoylation transcriptionally represses the target NR, but sumoylation of the orphan receptor RORα has been reported to induce transcriptional activity^25^. Moreover, some NRs such as liver receptor homolog-1 (LRH-1), liver X receptors (LXRs) and peroxisome proliferator-activated receptor γ (PPARγ) show ligand-dependent sumoylation^26^,^27^,^28^. In these cases, sumoylation prevents the removal of the NCoR co-repressor complex from the target gene promoter and results in transcriptional repression. It has also been recently shown that fibroblast growth factor-21 (FGF21) inhibits the sumoylation of PPARγ in white adipose tissue (WAT) to regulate lipid metabolism^29^. Previous studies of SF-1 post-translational modifications have focused on their roles in the regulation of steroidogenic genes in adrenal glands and gonads. However, the importance of these post-translational modifications in the VMH remains unknown.

In the present study, we investigated the role of SF-1 post-translational modifications, including sumoylation and uibiquitination, in the regulation of energy homeostasis. Our results demonstrate that serum starvation leads to a sumoylation-mediated, ubiquitin-dependent degradation of SF-1 and that sumoylation regulates the transcriptional activity of SF-1.

## Results

### Serum starvation induces ubiquitin-dependent degradation of hypothalamic SF-1

**Figure 1:**
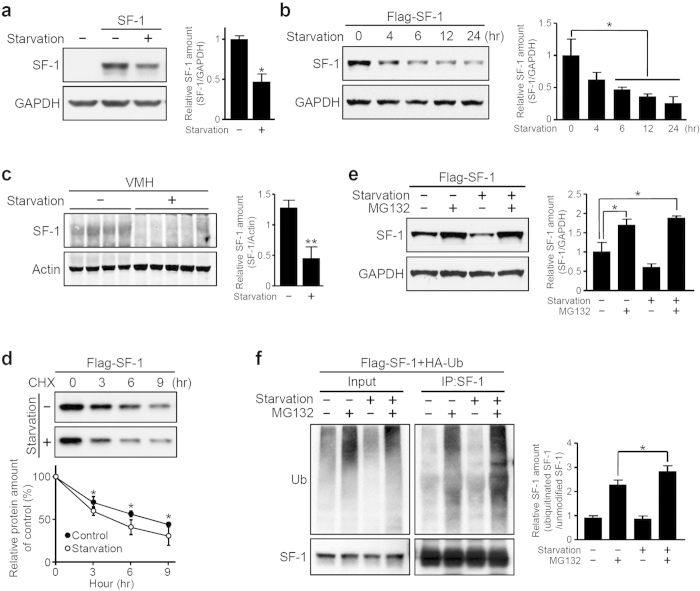
Serum starvation induces ubiquitin-dependent degradation of SF-1. (**a**) Decrease in SF-1 levels after 4 hours of serum starvation. HEK293 cells transfected with SF-1 were incubated in serum-free medium and analyzed by Western blot using an anti-SF-1 antibody (*P < 0.05). (**b**) Temporal regulation of SF-1 after starvation. SF-1-expressing cells were incubated in serum-free medium for the indicated times, and the SF-1 levels were analyzed by Western blot using an anti-Flag antibody (*P < 0.05). (**c**) Changes in SF-1 levels after serum starvation in organotypic VMH slices. Hypothalamic organotypic slices were incubated in standard medium or serum-free medium for 6 hours and then analyzed using an anti-SF-1 antibody (**P < 0.01). (**d**) Protein turnover of SF-1 under different nutritional conditions. HEK293 cells were transfected with Flag-tagged SF-1 and treated with CHX (30 mg/ml) with or without serum (*P < 0.05). (**e**) Degradation of SF-1 by the ubiquitin-dependent proteasome system. Cells were treated with or without MG132 at 25 μM for 6 hours in normal or serum starvation conditions (*P < 0.05). (**f**) Increased SF-1 ubiquitination in the serum-starvation condition. SF-1 was immunoprecipitated using an anti-SF-1 antibody followed by Western blot using either the anti-HA or anti-SF-1 antibodies. The values are presented as the mean ± SEM from three independent experiments (*P < 0.05).

We first set out to investigate the effect of nutritional conditions on SF-1 expression in the hypothalamus. To mimic a low nutrient fasting condition, we maintained HEK293 or Neuro2A cells in serum-free media after overexpression of SF-1. As shown in Fig. 1a,b and Supplementary Fig. S1a, SF-1 levels were significantly reduced by serum starvation and these changes were temporally regulated. To test whether SF-1 levels are similarly modulated by nutrient levels in the mouse brain, we established hypothalamic organotypic slice cultures and then incubated the tissues in serum-free media for 6 hours^30^. Consistent with the *in vitro* results, SF-1 expression was dramatically decreased in organotypic slices deprived of serum (Fig. 1c). These results strongly suggest that SF-1 is decreased during serum-starved conditions like fasting.

The regulation of protein levels by the ubiquitin-proteasome system has been well described^31^,^32^. Moreover, previous studies have been reported the importance of the ubiquitin system in the hypothalamic control of energy balance^33^,^34^,^35^. To determine whether the observed decrease in SF-1 levels is caused by protein degradation, we first measured the protein turnover of SF-1 during serum starvation in the presence of cycloheximide (CHX), an inhibitor of protein synthesis (Fig. 1d). We found that even in the presence of CHX, SF-1 levels decreased more rapidly in serum starvation conditions, suggesting that these changes were not a result of differences in protein synthesis. SF-1-expressing cells were then treated with MG132, an ubiquitin-dependent proteasome inhibitor (Fig. 1e and Supplementary Fig. S1b). The levels of SF-1 remained unchanged with MG132 treatment, regardless of the presence or absence of serum. These findings suggest that changes in SF-1 levels during starvation were due to ubiquitin-mediated degradation. To further confirm this, Flag-tagged SF-1 and HA-tagged ubiquitin were co-transfected into HEK293 cells. The cells were subjected to different nutrient conditions, with or without MG132, and immunoprecipitated using an anti-SF-1 antibody followed by Western blot using anti-HA or anti-SF-1 antibodies. As shown in Fig. 1f, treatment with MG132 markedly enhanced the ubiquitination of SF-1. Moreover, the ubiquitination signal of SF-1 was further intensified in the serum starvation condition compared to the normal condition. Taken together, these data demonstrate that starvation induces the ubiquitin-dependent degradation of SF-1.

### Sumoylation promotes degradation of SF-1

**Figure 2:**
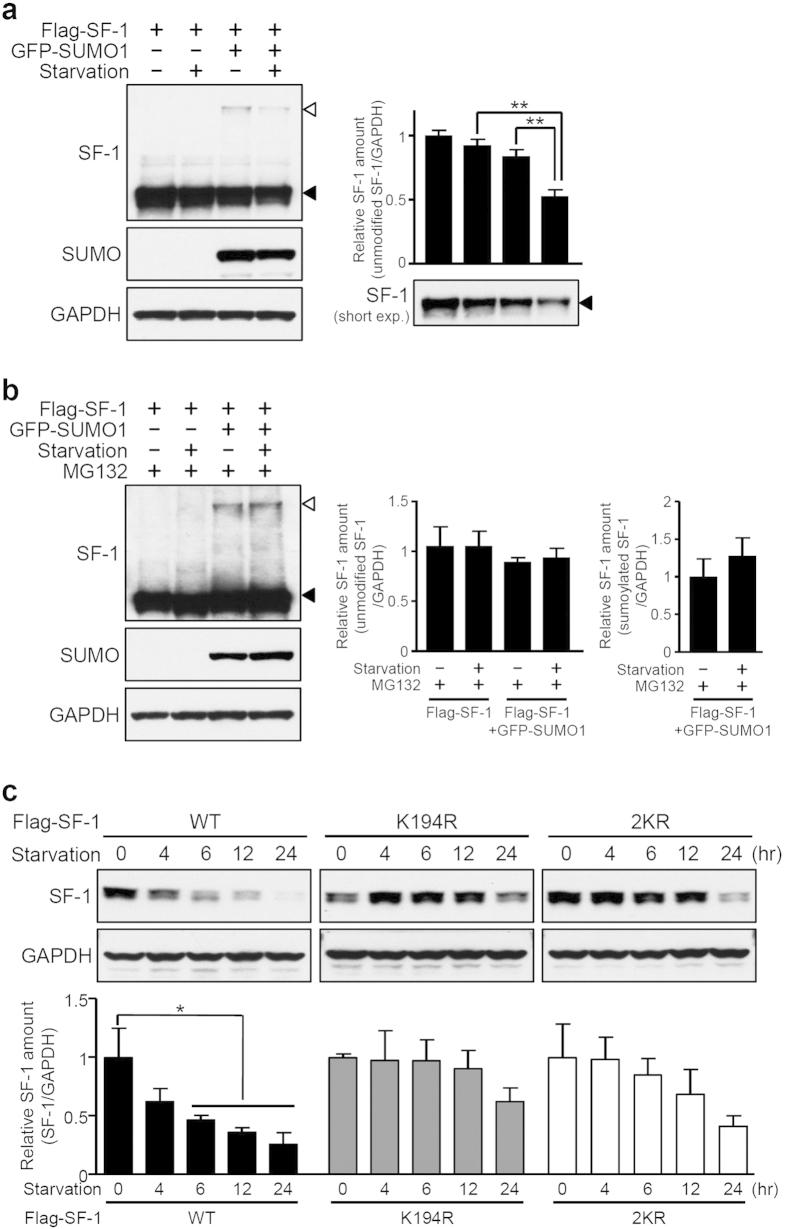
Sumoylation is required for degradation of SF-1. (**a**) Reduction of SF-1 sumoylation during serum starvation. HEK293 cells were transfected with Flag-SF-1 alone or together with GFP-fused SUMO1 and deprived of serum for 6 hours. Proteins were detected using anti-Flag or anti-GFP antibodies. Arrowheads indicate the unmodified SF-1 (black) and sumoylated SF-1 (white). The values are presented as the mean ± SEM from three independent experiments (**P < 0.01). (**b**) Proteasome inhibitor MG132 blocks SF-1 degradation. SF-1 and SUMO1-expressing cells were maintained with normal culture medium or serum-free medium together with MG132 at 25 μM for 6 hours. (**c**) Decreased starvation-induced degradation in sumoylation-defective SF-1 mutants. Flag-tagged SF-1 encoding wild-type (WT), the K194R mutant (K194R), or the K119/194R double mutant (2KR) were expressed in HEK293 cells and incubated under starvation conditions for the indicated times. The values are presented as the mean ± SEM from three independent experiments (*P < 0.05).

Ubiquitin often competes with small ubiquitin-related modifier (SUMO) to bind to the same target lysine residues; such that ubiquitination promotes proteasome-dependent proteolysis, while sumoylation stabilizes target proteins^36^,^37^,^38^,^39^. However, sumoylation can also be coupled with the ubiquitin-proteasome system to trigger ubiquitin-dependent degradation^40^,^41^,^42^. Because SF-1 has been previously shown to be modified by SUMO^19^,^23^, we sought to examine whether sumoylation is associated with starvation-induced SF-1 degradation. To this end, Flag-tagged SF-1 was transfected with or without GFP-fused SUMO1 into HEK293 cells and the cells were incubated in different nutritional conditions for 6 hours and analyzed by Western blot using anti-Flag or anti-GFP antibodies. As shown in Fig. 2a, both sumoylated SF-1 and unmodified SF-1 were decreased by serum starvation. Because the level of unmodified SF-1 in normal serum conditions was also slightly reduced when cells were transfected with GFP-SUMO1, we set out to test whether sumoylation directly affects SF-1 ubiquitination. We found that in cells that were treated with MG132, both the sumoylated and unmodified SF-1 were retained at the same levels regardless of nutritional conditions (Fig. 2b). These results suggest that sumoylation may promote the degradation of SF-1. To directly confirm this, we transfected wild-type SF-1 or sumoylation-deficient mutant constructs (K194R and 2KR) into either HEK293 or Neuro2A cells^19^ and removed serum for up to 24 hours. Consistent with our previous data, wild-type SF-1 levels were significantly decreased in a time-dependent manner; however, starvation-induced degradation of the mutant proteins was markedly blunted in both cell lines (Fig. 2c and Supplementary Fig. S2). These results indicate that sumoylation is required for the ubiquitin-dependent degradation of SF-1 under serum starvation conditions.

### Regulation of SF-1 transcriptional activity by sumoylation and ubiquitination

**Figure 3:**
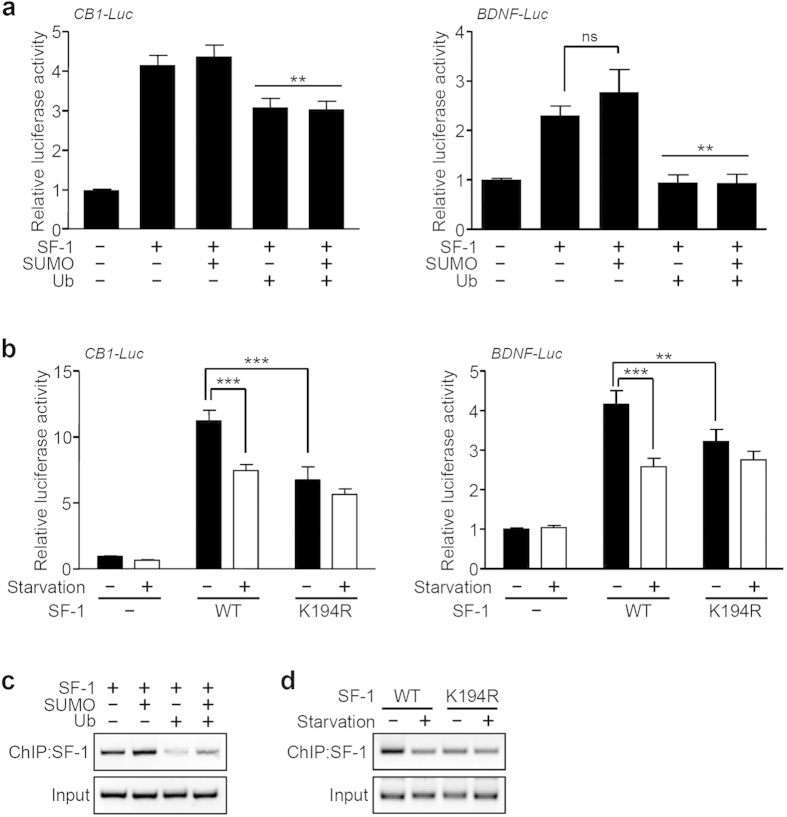
Transcriptional regulation of SF-1 by sumoylation and ubiquitination. (**a**) Effects of SUMO and ubiquitin on the regulation of transcriptional activity of SF-1. HEK293 cells were transfected with CB1-Luc, BDNF-Luc, SF-1, SUMO, or ubiquitin as indicated and luciferase activities were determined (**P < 0.01 vs. SF-1 only). (**b**) Transcriptional activity of wild-type SF-1 or the K194R mutant under the control of nutritional conditions. The values are presented as the mean ± SEM from more than three independent experiments (***P < 0.001, **P < 0.01). (**c**) SF-1 binding on CB1 promoter is modulated by sumoylation and ubiquitination. Chromatin was extracted from Neuro2A cells expressing Flag-SF-1, SUMO, or ubiquitin and immunoprecipitated using anti-Flag antibody. (**d**) Binding of wild-type SF-1 or the K194R mutant on the CB1 promoter under different nutritional conditions. Neuro2A cells were transfected with Flag-tagged SF-1 constructs encoding wild-type or the K194R mutant and incubated in culture medium with or without serum for 6 hours.

We next investigated the role of sumoylation and ubiquitination in the regulation of transcriptional activity of SF-1. To address this question, we employed luciferase constructs containing the SF-1 response element of the CB1 or BDNF promoters, which have previously been identified as downstream targets of SF-1 in the VMH^11^,^12^,^13^. The CB1- (CB1-Luc) or BDNF-luciferase reporter (BDNF-Luc) was transfected along with SF-1, SUMO, or ubiquitin into HEK293 cells and the luciferase activity was measured (Fig. 3a). As expected, SF-1 increased expression of the target genes. Co-expression of SUMO with SF-1 had no effect on SF-1-induced transcriptional activation, while co-transfection of ubiquitin significantly blunted luciferase activity (Fig. 3a). To examine whether these target genes were regulated by sumoylation under different nutritional conditions, we co-transfected the reporters with wild-type SF-1 or K194R mutant constructs and compared luciferase activity. As shown in Fig. 3b, serum starvation significantly reduced the ability of wild-type SF-1 to activate the transcription of CB1 or BDNF. On the other hand, cells co-transfected with K194R showed significantly and similarly reduced luciferase activity in both normal and starvation conditions. These results suggest that sumoylation of SF-1 is required for the regulation of SF-1 transcriptional activity and that the SF-1 activity is modulated by nutrient levels (Fig. 3b).

To examine whether the changes in nutrient-dependent transcriptional activity of SF-1 correlated with the ability of SF-1 to bind to the CB1 promoter, we performed chromatin immunoprecipitation (ChIP) assays. After transfection of Flag-tagged SF-1 with GFP-SUMO and/or HA-ubiquitin into Neuro2A cells, we performed immunoprecipitation using an anti-Flag antibody and the DNA was analyzed by PCR using primers specific for the CB1 promoter region, including SF-1 binding sites. SF-1 binding at the CB1 promoter was severely attenuated by ubiquitin alone or together with SUMO (Fig. 3c). Moreover, the K194R mutation as well as serum starvation inhibited the ability of SF-1 to bind to the CB1 promoter (Fig. 3d). Thus, these data clearly demonstrate that post-translational modifications of SF-1 such as sumoylation and ubiquitination are modulated by nutritional availability and play important roles in the regulation of target gene transcription.

### Starvation-induced SF-1 degradation is inhibited by insulin

**Figure 4:**
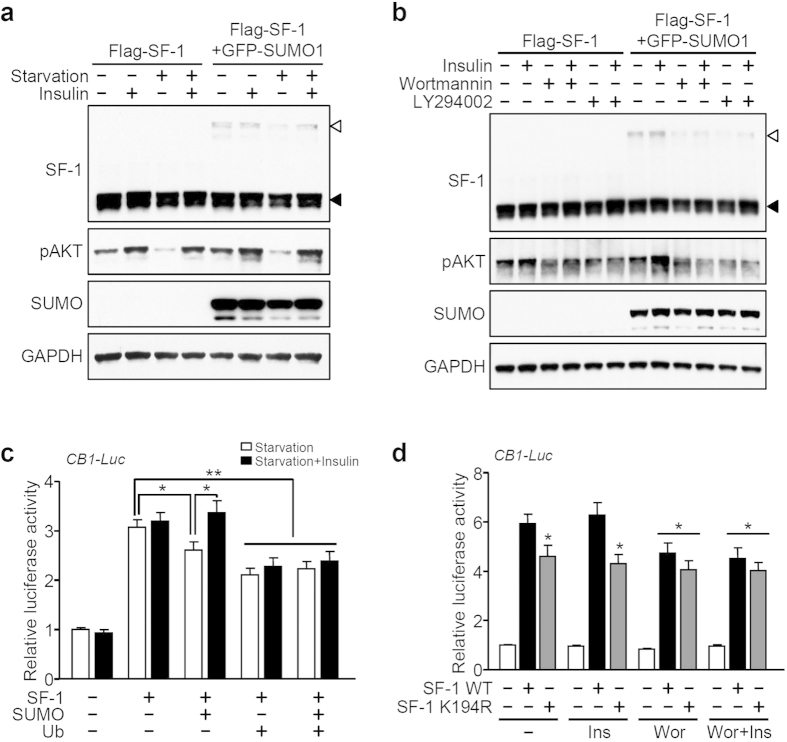
SF-1 stability and insulin signaling. (**a**) Attenuation of starvation-induced SF-1 degradation by insulin. HEK293 cells expressing Flag-SF-1 alone or together with GFP-SUMO1 were incubated with or without insulin (100 nM) under different nutritional conditions for 6 hours. Proteins were then observed using anti-Flag, anti-pAKT or anti-GFP antibodies. (**b**) Sumoylation-mediated degradation of SF-1 is regulated by the insulin-PI3K signaling pathway. Cells were treated with wortmannin (1 μM) or LY294002 (50 μM) for 1 hour and then incubated with vehicle or insulin (100 nM) for 5 hours. (**c**) SUMO-induced attenuation of SF-1 transcriptional activity under starvation conditions is restored by insulin. HEK293 cells were transfected with the CB1-Luc reporter and the indicated factors, and incubated in serum-free medium with or without insulin (100 nM) for 6 hours (*P < 0.05, **P < 0.01). (**d**) Involvement of PI3K signaling in the regulation of SF-1 transcriptional activity. SF-1-expressing cells were pre-treated with wortmannin (Wor, 1 μM) for 1 hour and then treated with insulin (Ins, 100 nM) for 5 hours under normal conditions. The values are mean ± SEM from more than three independent experiments (*P < 0.05 vs. SF-1 WT).

Many peripheral hormone levels are directly regulated in response to nutritional status. For instance, ingestion of food promotes the secretion of insulin, while fasting induces ghrelin expression. These hormones can act directly on the brain to regulate food intake and body weight homeostasis^43^,^44^. Previous studies have shown that insulin signaling via the phosphoinositide 3-kinase (PI3K) pathway in the VMH is critical for energy homeostasis^9^,^45^,^46^,^47^,^48^. Therefore, we hypothesized that insulin may be involved in the sumoylation and ubiquitination of SF-1. We first examined whether insulin has any effect on the stability of SF-1. Under normal conditions, the protein levels of both unmodified SF-1 and its sumoylated form were slightly intensified when cells were treated with insulin, and starvation-induced degradation of both forms was blunted by insulin treatment (Fig. 4a). Treatment with PI3K inhibitors such as wortmannin or LY294002 reduced the stability of both unmodified and sumoylated forms of SF-1 (Fig. 4b). These results imply that insulin-PI3K signaling may be associated with the regulation of the SF-1 function, especially in the control of sumoylation and ubiquitination.

To investigate the effect of insulin-PI3K signaling on the regulation of transcriptional activity of SF-1, we fasted cells with or without insulin and measured the transcriptional activation of CB1. Transcriptional activation of CB1 in serum starvation conditions was repressed in a similar fashion by co-expression of SUMO or ubiquitin (Fig. 4c). Importantly, in cells transfected with SUMO, the starvation-induced decrease in CB1 luciferase activity was rescued by insulin treatment. However, insulin did not effect luciferase activity when cells were co-expressed with ubiquitin alone or together with SUMO (Fig. 4c). Moreover, treatment with PI3K inhibitor blocked the effect of insulin on the CB1 transcriptional activation by wild-type SF-1, whereas insulin and wortmannin had no effect on the transcriptional activity of the K194R mutant (Fig. 4d). Collectively, these results strongly suggest that the insulin-PI3K pathway plays an important role in the regulation of stability as well as transcriptional activity of SF-1.

## Discussion

Recent work has highlighted the importance of hypothalamic SF-1 in energy homeostasis, particularly in the control of diet-induced thermogenesis through leptin and insulin signaling pathways. Mice with a VMH-specific SF-1 knockout show high-fat diet-induced obesity due to impaired thermogenesis and reduced energy expenditure. On the other hand, mice with a VMH-specific deletion of the upstream transcription factor FoxO1 overexpress SF-1 and remain lean when fed a high-fat diet due to increased energy expenditure. FoxO1 directly regulates SF-1 transcription by binding to the SF-1 promoter region^5^,^49^. Here, we found that the expression of SF-1 was decreased during serum starvation, while treatment with insulin blocked the starvation-dependent degradation and reactivated transcriptional activity of SF-1.

**Figure 5:**
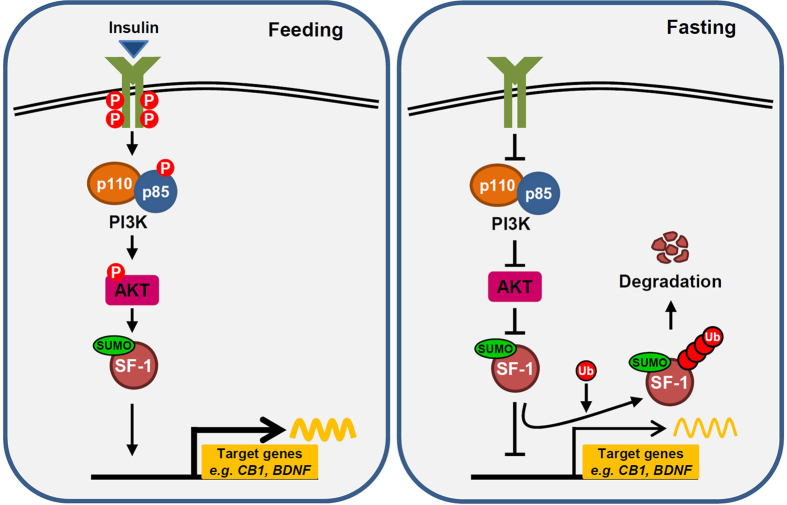
Model of the molecular mechanisms of SF-1 regulation by sumoylation and ubiquitination. In fed conditions, SF-1 sumoylation is increased by activation of the insulin-PI3K signaling pathway and promotes SF-1-mediated transcriptional regulation. Conversely, sumoylation of SF-1 acts as a degradation signal under fasted conditions. Therefore, sumoylation promotes ubiquitin-dependent degradation of SF-1 and attenuates SF-1 transcriptional activity under fasted condition.

Sumoylation of SF-1 is important for the development of endocrine tissues such as testes and adrenal glands and for its function as a transcription factor^19^,^22^,^23^,^50^. Previous studies have mostly focused on the role of SF-1 sumoylation in the regulation of steroidogenesis. In the present study, we showed that nutritional conditions can influence post-translational modifications of SF-1, including sumoylation and ubiquitination, and that these post-translational modifications play essential roles in the regulation of SF-1 transcriptional activity. Importantly, these nutrient-regulated modifications are dependent on insulin-PI3K signaling. During the fed state, sumoylation of SF-1 is enhanced by insulin-PI3K signaling and induces SF-1-mediated transcriptional activation. Indeed, insulin treatment increased the stability of both unmodified and sumoylated SF-1. Moreover, PI3K inhibitors, wortmannin or LY294002, reduced the protein abundance and transcriptional activity of SF-1 in normal serum conditions. In contrast, in the fasting condition, sumoylation functions as a degradation signal and promotes ubiquitin-dependent degradation as well as inhibits transcription of target genes. Overexpression of ubiquitin repressed SF-1 transcriptional activity regardless of the nutritional conditions, wehereas overexpression of SUMO led to activation of target genes during the fed state or inhibition during starvation. This model underlying the modulation of SF-1 in different nutritional states is outlined in Fig. 5.

Sumoylation and ubiquitination are both involved in the regulation of downstream target gene expression^51^,^52^. For example, sumoylation up-regulates target gene transcription and promotes ubiquitin-dependent degradation of EGR1 and BMAL1. On the other hand, ubiquitination can either activate or inactivate downstream gene expression. EGR1 ubiquitination attenuates its transcriptional activity^53^,^54^, whereas ubiquitination of BMAL1 significantly stimulates target gene transcription^42^,^55^. Our study shows that SUMO and ubiquitin differentially regulate the transcriptional activity of SF-1. SUMO activated SF-1 transcriptional activity, while ubiquitin blocked the activity even in the presence of SUMO (Fig. 3a). Starvation, which triggers ubiquitin-dependent degradation of SF-1, also suppressed the luciferase activity of the CB1 and BDNF promoters. Mutations which block the sumoylation site have repressed transcriptional activation, regardless of nutritional status (Fig. 3b). These data demonstrate that sumoylation is a nutrient-sensitive regulator of SF-1 activity that may play an important role in maintaining the balance between transcriptional regulation and degradation of SF-1. Consequently, this nutrient-dependent post-translational modification directly affects the ability of SF-1 to regulate energy homeostasis.

Our data suggests that activation of insulin signaling plays a protective role for starvation-induced SF-1 degradation (Fig. 4). It has been demonstrated that the transcription factor FoxO1 is a negative regulator of SF-1 transcription, as well as a downstream target of insulin-PI3K signaling^46^,^49^,^56^. FoxO1 is localized in the nucleus and represses transcription by binding to the promoter regions of target genes, including SF-1. When cells are stimulated by insulin, PI3K induces AKT phosphorylation, which in turn leads to the phosphorylation and translocation of FoxO1 to the cytoplasm, and the transactivation of target genes. Indeed, we found that the activation of AKT was significantly blunted when cells were deprived of serum (Fig. 4a), suggesting that the SF-1 transcription may be attenuated by the sequestration of FoxO1 in the nucleus. These observations raise the possibility that the control of SF-1 by insulin signaling could be regulated by FoxO1-mediated SF-1 transcriptional regulation as well as post-translational modifications of SF-1.

Environmental stressors such as heat shock, oxidative and osmotic stress can affect the SUMO modification of some proteins^57^,^58^. We therefore suggest that nutrient conditions may also regulate sumoylation of proteins and ultimately lead to functional changes of such proteins. Indeed, a recent study has demonstrated that nutrient deprivation leads to sumoylation of sterol regulatory element-binding protein 1c (SREBP1c) via the glucagon pathway, resulting in the regulation of lipid metabolism^59^. Fasting promotes the phosphorylation of SREBP1c by protein kinase A (PKA), which then induces its sumoylation and ubiqitin-dependent degradation, repressing target gene transcription, and ultimately, decreasing lipogenesis. This observation is consistent with our finding that starvation enhances sumoylation-mediated degradation of SF-1 and regulates the transcriptional activity of SF-1.

In conclusion, our findings demonstrate a nutrient-dependent dual role of sumoylation for energy homeostasis and provide new insights of SF-1 regulation by post-translational modifications in the brain. Our data suggest that sumoylation and ubiquitination of SF-1 are crucial for the control of energy balance via the regulation of both protein stability and transcriptional activity of SF-1 under different nutritional conditions, supporting the importance of a diet-induced regulation of SF-1.

## Methods

### Cell culture and transfection

HEK293 and Neuro2A cells were cultured in Dulbecco’s modified Eagle’s medium (DMEM) supplemented with 10% fetal bovine serum (FBS) and 100 U/ml penicillin-streptomycin (Invitrogen) at 37 °C under 5% CO_2_. For transient transfection, HEK293 cells were seeded in 12-well plates the day before transfection and transfected with specific DNAs using Lipofectamine 2000 reagents (Invitrogen). One day after transfection, cells were incubated in serum-free medium and/or treated with drugs such as insulin (100 nM, Sigma), wortmannin (1 μM) and LY294002 (50 μM, Cayman Chemical) for 6 hours and then cells were lysed and subjected to Western blot or immunoprecipitation.

### Immunoprecipitation and Western blot

HEK293 cells were plated in 6-well plates and then incubated with Flag-tagged SF-1 and HA-tagged ubiquitin. One day after transfection, cells were treated with MG132 at 25 μM for 6 hours in the presence or absence of serum. Cells were harvested in RIPA buffer (50 mM Tris-HCl (pH 8.0), 150 mM NaCl, 1% Triton X-100, 1 mM EDTA, 1 mM EGTA, 0.5% sodium deoxycholate, 1× protease inhibitor cocktail and 1× phosphatease inhibitor cocktail (Roche)) and centrifuged at maximum speed for 20 min at 4 °C. Equal amounts of total protein were incubated with 2 μl anti-SF-1 specific antiserum^12^ overnight at 4 °C and then added to a protein G-Sepharose bead slurry for 2 hours at 4 °C. The final immune complexes were analyzed by Western blot.

For Western blots, proteins were resolved on 4–12% gradient SDS-PAGE gels (Invitrogen) and transferred to Nitrocellulose membranes (Bio-Rad). Target proteins were detected using anti-SF-1^12^, anti-HA (F-7) or anti-GAPDH (Santa Cruz Biotechnology), anti-Flag, anti-GFP or anti-Actin (Sigma), or anti-phospho-AKT (S473, Cell Signaling) antibodies. The immune complexes were visualized with an Enhanced Chemiluminescence (ECL) detection kit (Pierce) or an Odyssey IR imaging system (LI-COR Biosciences).

### Hypothalamic organotypic slice culture

The hypothalamic slices were made as previously described^60^. The brains were obtained from 9 to 11-day-old C57BL/6J mouse pups. Hypothalamic tissues were sectioned at a thickness of 250 μm on a vibratome (VT1000 S, Leica) in chilled Gey’s Balanced Salt Solution (Sigma) enriched with glucose (0.5%) and KCl (30 mM). The coronal slices containing the VMH region were placed on Millicell-CM filters (pore size 0.4 μm, diameter 30 mm, EDM Millipore) and then maintained at an air-media interface in MEM (Invitrogen) supplemented with heat-inactivated horse serum (25%, Invitrogen), glucose (32 mM) and GlutaMAX (2 mM, Invitrogen). The cultured slices were typically maintained for 10 days in standard medium, which was replaced two or three times a week. For experiments, the slices were incubated in standard medium (25% horse serum) or serum-free medium for 6 hours. Only the VMH regions from the slices were collected, and the samples were analyzed by Western blot.

### Luciferase reporter assay

To prepare BDNF reporter plasmid, a Bdnf IV fragment containing the SF-1 response element was generated by PCR using the previously described construct as a template^13^ and inserted into the pGL3-Promoter vector (Promega). For luciferase assays, HEK293 cells were seeded in 96-well plates and transfected with the indicated DNAs such as CB1-Luc reporter, BDNF-Luc reporter, SF-1, SUMO1 or ubiquitin using Lipofectamine 2000 reagents (Invitrogen). After 24 hours, cells were incubated in the culture medium with or without serum for 6 hours. To confirm the effects of insulin-PI3K signaling on the regulation of CB1 transcription, we treated the cells with wortmannin at 1 μM for 1 hour and then incubated with insulin at 100 nM for 5 hours. Cells were then lysed and the luciferase activity was measured using the Dual-Glo Luciferase Assay System (Promega) according to the manufacturer’s protocol. The firefly luciferase activity was normalized to Renilla luciferase.

### ChIP assays

Chromatin immunoprecipitation (ChIP) assays were performed according to the manufacturer’s instructions (EDM Millipore). SF-1 (WT or K194R mutant), SUMO1, or ubiquitin were expressed in Neuro2A mouse neuroblastoma cells, and the cells were incubated in the culture medium with or without serum for 6 hours. Cells were cross-linked with 1% formaldehyde for 10 minutes and each cross-linked sample was immunoprecipitated using 2 μg of anti-Flag (Sigma) antibody, and the DNA was purified by phenol-chloroform extraction and ethanol precipitation. PCR primers for the CB1 promoter spanned the proximal region of the SF-1 binding site of the CB1 promoter, 5′-CAGATCCCTTGGCGGAGT-3′ and 5′-CTTCGTTCTCCGGCTCTC-3′.

### Statistical analysis

The data are presented as means ± SEM. Comparison between multiple groups were determined for statistical significance by one-way ANOVA and Student’s t-test, and all data were tested for normal distribution. *P* < 0.05 was regarded as a statistical significance difference.

## Additional Information

**How to cite this article**: Lee, J. *et al.* Nutritional conditions regulate transcriptional activity of SF-1 by controlling sumoylation and ubiquitination. *Sci. Rep.* **6**, 19143; doi: 10.1038/srep19143 (2016).

## Supplementary Material

Supplementary Information
