# Supplementary material for: Nutritional conditions regulate transcriptional activity of SF-1 by controlling sumoylation and ubiquitination
Source: Sci Rep. 2016 Jan 11;6:19143. doi: 10.1038/srep19143 (PMC4707483; doi:10.1038/srep19143)
Supplement: Supplementary Information [file srep19143-s1.pdf]

Supplementary information

Nutritional conditions regulate transcriptional activity of SF-1 by controlling sumoylation and ubiquitination.

Jiwon Lee[1,†], Dong Joo Yang[2], Syann Lee[1], Gary D. Hammer[3], Ki Woo Kim[2] & Joel K. Elmquist[1]

[1]Departments of Pharmacology and Internal Medicine, Division of Hypothalamic Research, University of Texas Southwestern Medical Center, Dallas, Texas 75390, USA, [2]Departments of Pharmacology and Global Medical Science, Institute of Lifestyle Medicine and Nuclear Receptor Research Consortium, Wonju College of Medicine, Yonsei University, Wonju, 26426, South Korea, [3]Endocrine Oncology Program, Center for Organogenesis, University of Michigan Health System, Ann Arbor, Michigan 48109, USA. [†]Current address: Department of Anatomy and Cell Biology, Samsung Biomedical Research Institute, Sungkyunkwan University School of Medicine, Suwon, 16419, South Korea.

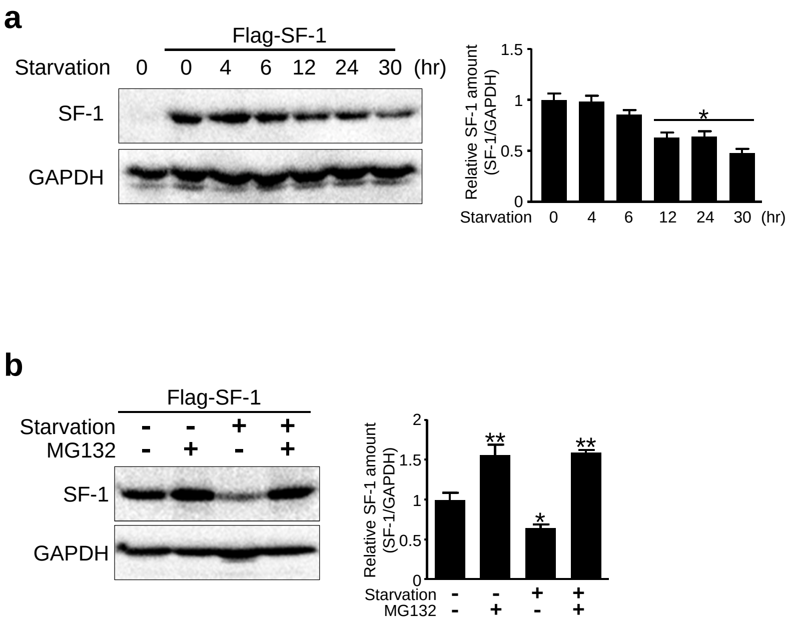

Supplementary Figure S1. Serum starvation induces ubiquitin-dependent degradation of SF-1 in Neuro2A cells. (a) Temporal regulation of SF-1 after starvation (**P* < 0.05). (b) Proteasome inhibitor MG132 blocks SF-1 degradation. MG132 was treated at 25 μM for 12 hours. The values are presented as the mean ± SEM from three independent experiments (**P* < 0.05, ***P* < 0.01 vs. control group).

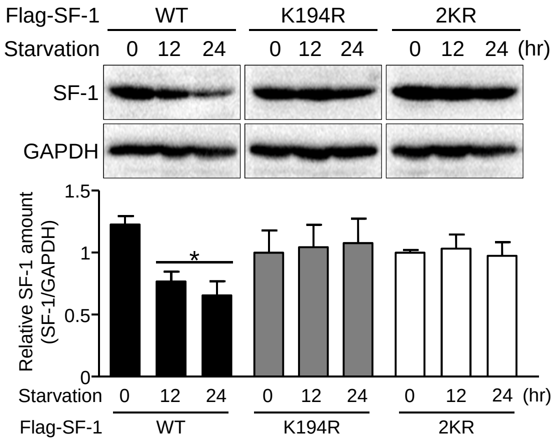

Supplementary Figure S2. Serum starvation induces sumoylation-dependent degradation of SF-1 in Neuro2A cells. Decreased starvation-induced degradation in sumoylation-defective SF-1 mutants. The values are presented as the mean ± SEM from three independent experiments (**P* < 0.05).

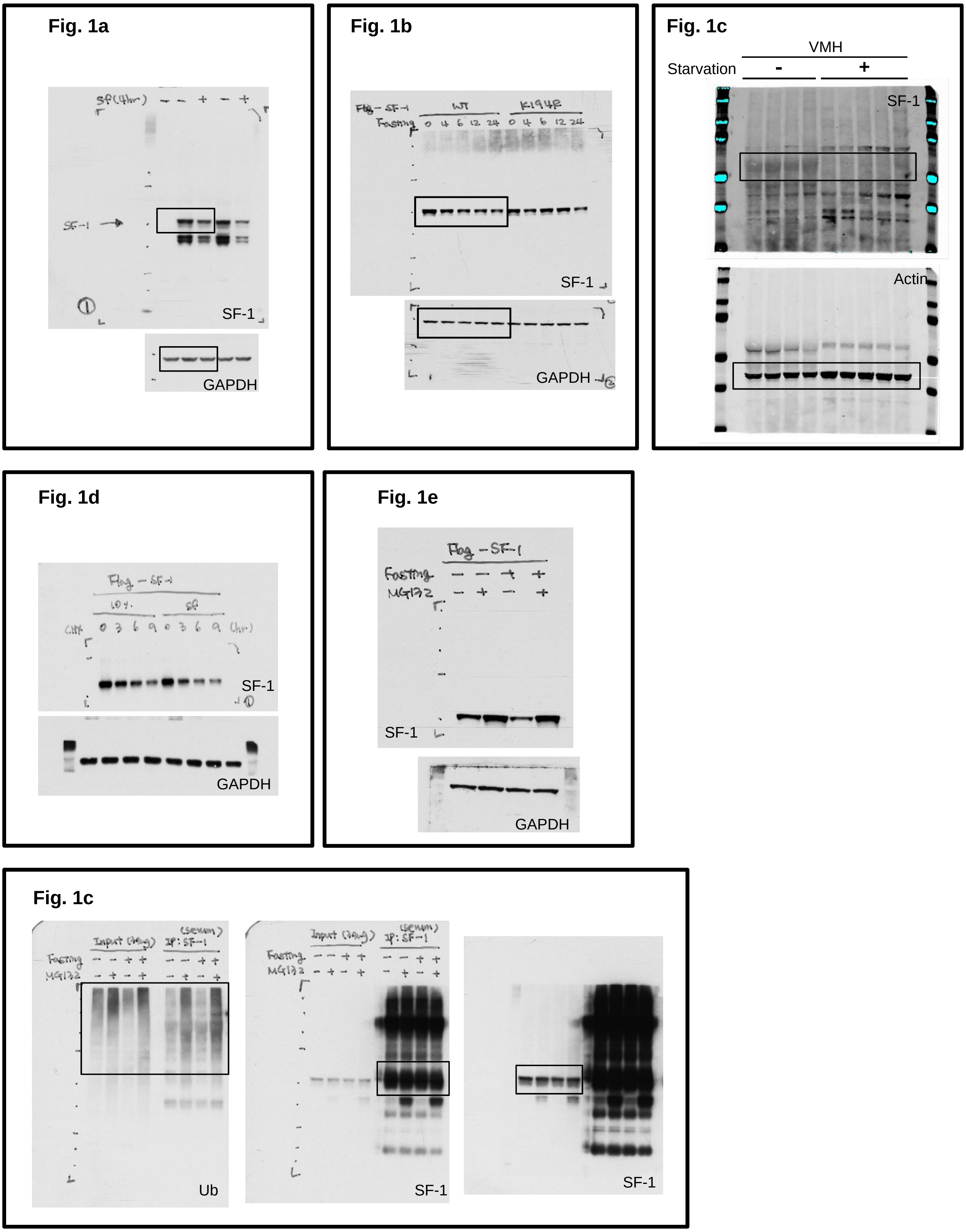

Supplementary Figure S3. Uncropped Western blot for the data in Figure 1.

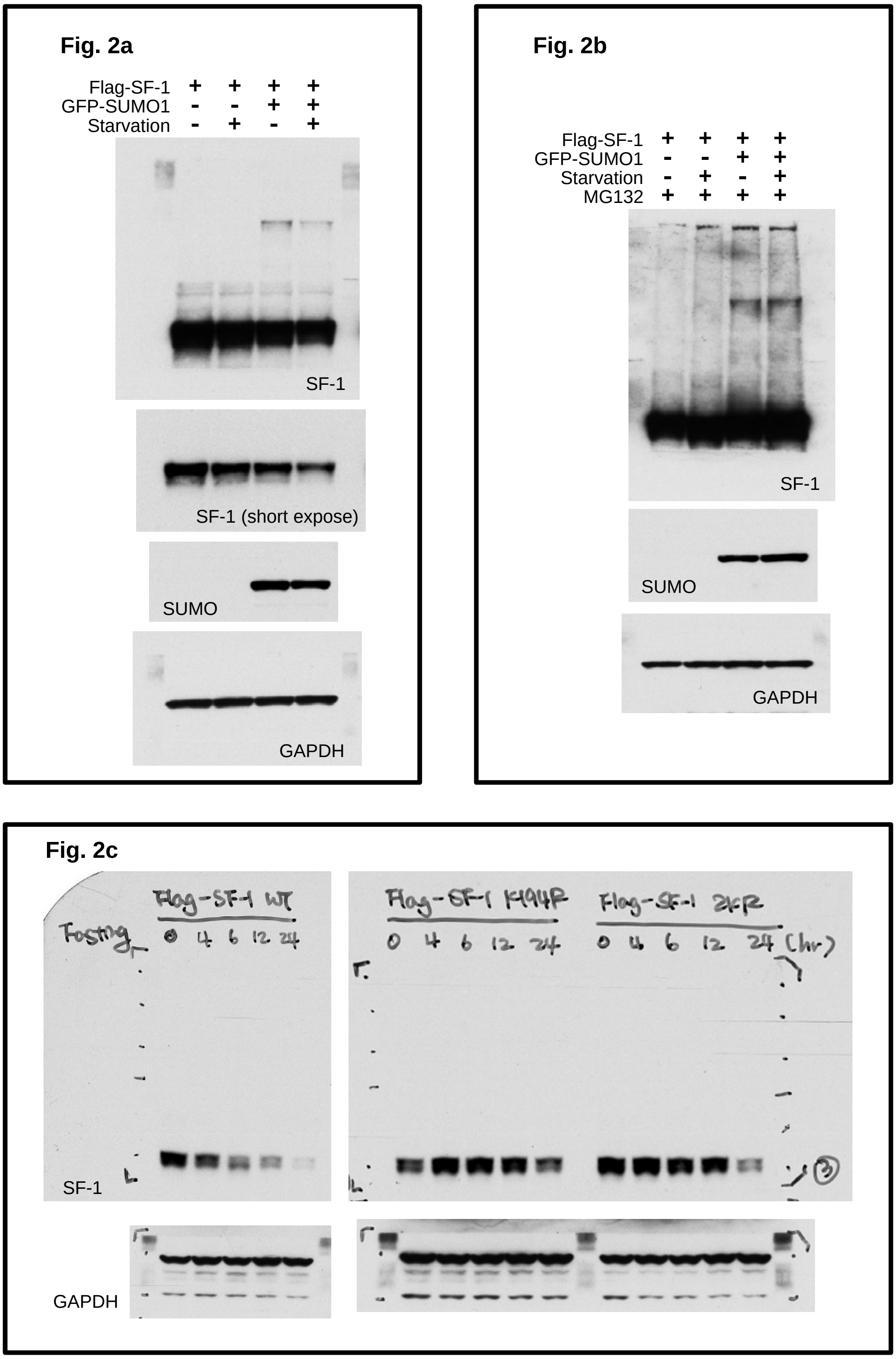

Supplementary Figure S4. Uncropped Western blot for the data in Figure 2.

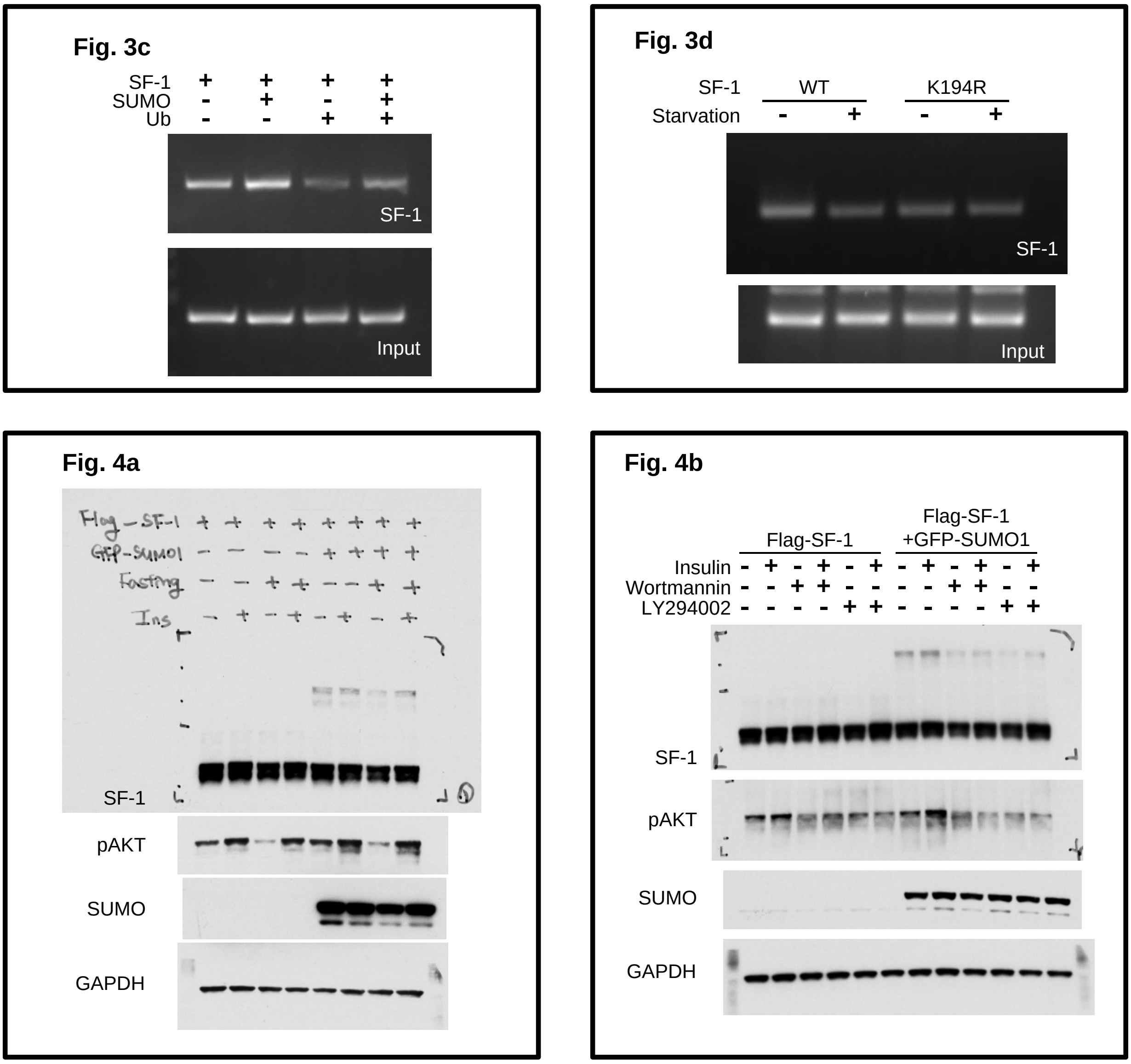

Supplementary Figure S5. Uncropped gel image and Western blot for the data in Figure 3 and 4.
